# Subjekt, Statistik, Wissenschaft: Epistemologische Positionierungen und Evidenzpraktiken in der klinischen Medizin seit 1949: Einleitung

**DOI:** 10.1007/s00048-021-00317-4

**Published:** 2021-10-29

**Authors:** Hans-Georg Hofer, Volker Roelcke

**Affiliations:** 1 Institut für Ethik, Geschichte und Theorie der Medizin, WWU Münster, Münster, Deutschland; 2 Institut für Geschichte der Medizin, Justus-Liebig-Universität Gießen, Gießen, Deutschland

Die Diskussionen zur Produktion und Validierung von klinisch relevantem und zuverlässigem Wissen sind seit den 1990er Jahren untrennbar mit der *Evidence based Medicine* (EbM) verknüpft. Die EbM ist seitdem auch in den Fokus historisch-epistemologischer, medizintheoretischer und wissenschaftsphilosophischer Arbeiten gerückt (siehe z. B. Daly 2005; Solomon 2015; Raspe 2018; Borck 2020). In Auseinandersetzung mit der EbM – und teilweise in deren Sog – richtete sich der Fokus auf die Hierarchisierung, Priorisierung, Rationalisierung oder Standardisierung medizinischen Wissens (Timmermans & Berg 2003), auf die *longue durée* medizinischer Quantifizierung und statistischer Verfahren in der Klinik (Weisz 2005) oder auf die Implementierung verlässlicher methodischer Standards in der Arzneimittelforschung (Marks 2009). Der Aufstieg der EbM selbst war dabei – wie Jones und Podolsky am Beispiel der parallelen Begriffskarriere des *Gold Standard* gezeigt haben – von paradoxen, zum Teil gegenläufigen Entwicklungen in Ökonomie und Medizin geprägt (Jones & Podolsky 2015). In jüngster Zeit sind die kontrovers geführten Debatten zur EbM auch zu einem Thema medizinischer Gegenwartsgeschichte geworden. Die Rede von einer „Krise der EbM“ (Bolt & Huisman 2018) zeigt, dass hierbei sprachliche Topoi bemüht werden, die ihrerseits in längeren Reflexionstraditionen stehen.

In der deutschsprachigen Medizingeschichte geht der Begriff der Evidenz nicht nur deutlich über die EbM hinaus, sondern auch viel weiter zurück – und weist zudem eine produktive semantische Spannung auf. Während der ins Deutsche übersetzte angelsächsische Evidenzbegriff der *Evidence based Medicine* im Sinne von Beleg, Indiz, Beweis verwendet wird – ähnlich dem Beweismittel in einem Gerichtsverfahren (*judicious use*, Sackett et al. 1996) –, meint Evidenz in der kontinentalen, auch deutschen philosophischen Tradition etwas anderes, nämlich eine Erkenntnis, die sich unmittelbar aus dem Augenscheinlichen ergibt; eine offenkundige, einleuchtende Gewissheit. Die konträren Bedeutungen der beiden Evidenzbegriffe mögen in der Rezeption der EbM irritierend gewirkt und manches Missverständnis nach sich gezogen haben, doch liegt in diesem Umstand eine große Chance und ein epistemisches Potenzial gerade für wissenschaftshistorische Untersuchungen, da der Fokus weit geöffnet werden kann, vorschnelle Festlegungen vermieden und Denkzusammenhänge hergestellt werden können. In einer solchen breiten Perspektive auf den Begriff der Evidenz haben Mehrdeutigkeiten und methodische Vielfalt ihren Platz und ihre Berechtigung. Eine solche Position steht auch nicht im Widerspruch zu Stimmen aus der rezenten wissenschaftlichen Medizin selbst („Evidenzen-basierte Medizin“, Raspe 2001; „diversity of approaches“, Rawlins 2008; „triangulation of evidence“, Munafò & Smith 2018). Zu fragen wäre in einer solchen Perspektivierung, wer in welchen Kontexten genau welche spezifische Position zur Begründung von relevantem und validem Wissen vorgebracht hat, und ebenso, ob und in genau welcher Form in solchen Begründungsstrategien der Begriff Evidenz oder möglicherweise andere basale epistemologische Begriffe verwendet wurden. Weiter wäre zu fragen, in welcher Weise und veranlasst durch welche Faktoren sich die Positionen relevanter individueller Akteure oder „Denkkollektive“ über die Zeit veränderten und in welche Praktiken der Plausibilisierung oder Evidenzproduktion die verschiedenen Positionen eingebunden waren. Schließlich erlaubt das Privileg der Distanz, die durch den historischen Blick ermöglicht wird, auch die Berücksichtigung der Frage, ob in spezifischen historischen Positionen neben (oder anstatt) dem herkömmlichen Fokus auf der wissenschaftlichen Rechtfertigung einer abstrakt-theoretischen Aussage („warrant of theory“) möglicherweise auch eine alternative Sichtweise möglich war, die nach den Rechtfertigungen für die konkreten Anwendungen von neuem wissenschaftlichen Wissen fragt („evidence for use“), das heißt, nach den vorgesehenen Zielen einer Anwendung, den praktischen Voraussetzungen und dem Geltungsbereich von Validierungsprozessen, den eingesetzten Mitteln, absehbarem Nutzen und Risiken (für eine wissenschaftsphilosophische Begründung einer solchen Sichtweise vgl. Cartwright 2006).

Diesen Fragen soll in der vorliegenden *Special Section* exemplarisch anhand von intensiven Debatten im Grenzgebiet zwischen Innerer Medizin, Psychosomatik und Psychiatrie in den mittleren Jahrzehnten des 20. Jahrhunderts nachgegangen werden. Fokus und Bühne für diese Debatten war der 1949 veranstaltete Kongress der Deutschen Gesellschaft für Innere Medizin:^1^ Im Gründungsjahr der Bundesrepublik an den angestammten Ort der jährlich veranstalteten Zusammenkünfte in Wiesbaden zurückgekehrt, lag er exakt an der Gelenkstelle zwischen Nachkriegszeit und demokratischem Neuanfang. Im Gedächtnis blieb der Kongress vor allem durch die ebenso leidenschaftlich wie elegant ausgetragene Kontroverse um die „Psychosomatische Medizin“. Von ihren Proponenten sowohl auf programmatischer wie auf methodischer Ebene in einen Gegensatz zur „naturwissenschaftlichen Medizin“ gebracht, wurden in der Diskussion grundsätzliche epistemologische Auffassungsunterschiede sichtbar, die über den Kongress hinaus zur Reflexion und Positionierung herausforderten. Die 1949 kulminierenden Kontroversen standen in längeren Kontinuitäten. Erste Konturen der konfligierenden Positionen zeigen sich bereits in den 1920er Jahren – und wirkten über den Kongress hinaus bis in die 1970er Jahre fort.

Die Grundsätzlichkeit und Differenziertheit, mit der diese Debatten geführt wurden, haben mit dem spezifischen historischen Kontext zu tun. In seinem äußeren Rahmen stand der Kongress für Tradition und Aufbruch, für eine Rückkehr zur Normalität nach Krieg und Besatzungszeit. Auf der Bühne zeigte sich jedoch eine Medizin, die in ihrer wissenschaftlichen Identität verunsichert war und – in den Worten des Internisten Paul Martini – „ihre Fundamente erzittern fühlte“ (Martini 1948: 3). Der Nürnberger Ärzteprozess, der menschenverachtendes Verhalten auch von renommierten Ärzten an die Öffentlichkeit gebracht hatte, lag 1949 gerade erst zwei Jahre zurück. Die Konfrontation mit dieser drastischen Realität führte zwar häufig zu den in der historischen Forschung mittlerweile breit dokumentierten Verhaltensweisen von Negation, Schweigen und Exkulpierung (Roelcke et al. 2014), doch ließ sie auch eine tiefe Sorge, eine epistemologische Unruhe entstehen, die fachöffentlich gerade auch von jenen Ärzten zum Ausdruck gebracht wurde, die auf dem Kongress auftraten: Worin lagen die Ursachen und Voraussetzungen der dokumentierten medizinischen Unrechtstaten? Hatte sich – so argumentierten etwa Viktor von Weizsäcker, Alexander Mitscherlich, Paul Martini, Arthur Jores und Thure von Uexküll in ähnlicher Art und Weise – in den Medizinverbrechen nicht ein ganz grundsätzliches Problem manifestiert – und zwar in Gestalt von destruktiven Potenzialen, die in der Struktur der modernen, naturwissenschaftlich geprägten Medizin selbst angelegt waren? Gingen von Materialismus und Reduktionismus, von Experiment und Objektivierung bislang übersehene Gefährdungen aus? Wo waren Korrekturen vorzunehmen, und nach welchen Kriterien und mit welchem Ziel? Welche Forschungsmethoden und Theorien waren in der Psychosomatischen Medizin, überhaupt in der klinischen Medizin geeignet, ein wirksames und verantwortliches Handeln am kranken Menschen zu realisieren? Fragen wie diese standen teils im Hintergrund, teils im Vordergrund der methodischen Kontroversen, verliehen ihnen Tiefgründigkeit, Intensität und Schärfe. Ihnen gemeinsam war die Sorge um das epistemologische Selbst der Medizin.

Die nachfolgenden Beiträge eint der Versuch, den Begriff der Evidenz nicht vorauszusetzen, sondern in seinen historischen Varianzen und Ausprägungen aufzuspüren und zu reflektieren.^2^ Dabei soll deutlich werden, dass die Frage nach der wissenschaftlichen Evidenz nicht aus sich selbst beantwortet werden kann, sondern konkreter methodischer Begründungsmodi und Erzeugungspraktiken bedarf und damit von epistemologischen Situierungen, Kontexten und Zielvorstellungen abhängig ist.

Hans-Georg Hofer zeichnet zunächst die Konturen des Kongresses und leuchtet seinen spezifischen historischen Kontext aus. Im Mittelpunkt seines Beitrags steht einer der zentralen Akteure der klinisch-internistischen Medizin, Paul Martini. Auf dem Kongress trat Martini als exponierter Kritiker der Psychosomatischen Medizin in Erscheinung. Auf Basis seiner Arbeiten zur Methodologie der klinisch-therapeutischen Forschung insistierte er auf dem (mechanistisch-probabilistisch geprägten) Kausalitätsbegriff in der Krankheitsentstehung sowie auf statistisch abgestützten Evidenzpraktiken in der Klinik. Damit koppelte Martini die Wirksamkeit therapeutischer Interventionen an das Erbringen von klinischen „Beweisen“ und rückte den Begriff der Evidenz in Richtung einer evaluativen Dimension. Die Rekonstruktion von Martinis Positionierungen zeigt, dass die Konfliktlinien eine längere Vorgeschichte haben, die etwa mit Blick auf Viktor von Weizsäcker, den ersten Hauptvortragenden auf dem Kongress, bis in die 1930er Jahre zurückreichten. Rekonstruiert werden weiterhin die von dem Kongress ausgehenden Forschungsbemühungen Martinis, eine Methodologie der Psychosomatischen Medizin *sensu stricto* auszuarbeiten – ein Vorhaben, das allerdings Fragment blieb.

Mit einem Perspektivenwechsel auf Alexander Mitscherlich und seine 1949 markant sichtbare Rolle als Proponent der Psychosomatischen Medizin leitet Steffen Dörre seinen Beitrag ein. Mitscherlich stand 1949 mit seiner konsequenten methodologischen Orientierung am Subjekt sowie seiner Kritik an den Limitationen statistischer Evidenzerzeugung in einem scharfen Gegensatz zu Martini. Die kontroversen Auseinandersetzungen auf dem Kongress fanden in den darauffolgenden Jahren mehrere Neuauflagen. Dörre zeichnet ein feinkörniges Bild von Mitscherlichs fortgesetzten epistemologischen Neupositionierungen, die anthropologisch-biografisch, tiefenpsychologisch-psychoanalytisch sowie später auch sozialpsychologisch und soziologisch grundiert waren. Gemeinsamer Nenner blieb die mit insistierender Schärfe vorgetragene Grundsatzkritik an „der naturwissenschaftlichen Medizin“ sowie das Plädoyer, ein am Menschen orientiertes Krankheitsverständnis auszuarbeiten und über erzählte Erfahrungen in der Medizin zu etablieren. Mitscherlichs Forderung, Krankheit als Geschichte zu verstehen, wirkt im Hinblick auf rezente Überlegungen der Medical Humanities oder Narrative Medicine erstaunlich aktuell – und wäre eine eingehendere Betrachtung wert.

Maike Rotzoll unternimmt einen weiteren Standortwechsel und untersucht, wie sich 1949 exponierte Vertreter der westdeutschen Psychiatrie zur Psychosomatischen Medizin stellten. Dabei kann sie zeigen, dass die drei psychiatrischen Sprecher auf dem Kongress jeweils unterschiedliche methodische Auffassungen und Evidenzpraktiken vertraten. Ihr Spektrum reichte von der Objektivierung neuropathologischer Organbefunde über die Anwendung von Statistik bis zur Inanspruchnahme der klinischen Erfahrung. Die Frage nach dem Subjekt und seinem Status im psychiatrischen Wissenschaftsverständnis ließ insbesondere den Frankfurter Ordinarius Jürg Zutt nicht mehr los. Er zentrierte in seinen methodischen Überlegungen die Erfahrung des Klinikers, die ihm „auf dem Wege zu einer anthropologischen Psychiatrie“ unverzichtbar erschien. Sein Beispiel zeigt weiterhin, dass der Begriff „anthropologisch“ nach einer kurzen Latenzzeit erneut Auftrieb bekam und neue Anverwandlungen erfuhr. Was „anthropologisch“ meinte, wer diesen Begriff in welcher Absicht verwendete und wie sich dessen Bedeutungsidentitäten wandelten, wäre in weiterer Forschung zu klären.

Im Beitrag von Volker Roelcke wird eine längerfristige Nachwirkung der Kongressdebatten in den Blick genommen, nämlich ein Forschungsprojekt des Internisten und Psychosomatikers Thure von Uexküll zur Ätiologie und Pathogenese der Hypertonie. Dieses Projekt verwendete eine komplexe Versuchsanordnung, in der individual- und sozialpsychologische Faktoren mit somatischen Parametern integriert betrachtet und, wie von Martini gefordert, auf Kausalbeziehungen hin untersucht wurden. Gleichzeitig hatten die Deutungen der Patienten einen privilegierten Status in diesem Experimentalsystem. Empirische Belege, Nachvollziehbarkeit und Reproduzierbarkeit – ebenfalls Anforderungen, die Martini 1949 formuliert hatte – waren für Uexküll elementare Kriterien für relevantes und valides Wissen. Die im Kontext des Forschungsprojekts entwickelten Konzepte des „Motivzusammenhangs“, der „Situation“ und des „emotionalen Programms“ erlaubten es Uexküll, zentrale Komponenten und die Dynamik beim Ineinandergreifen von Körperlichem und Seelischem zu beschreiben und für die medizinische Forschung und Praxis handhabbar zu machen. Das darauf aufbauende Konzept des „Situationskreises“ wurde in der Folge zur theoretischen Grundlage von Uexkülls Lehrbuch der Psychosomatischen Medizin.

In der Zusammenschau verstehen sich die Artikel als Beitrag zur Freilegung und Neuentdeckung jener Debatten, die bei der Konstituierung von epistemologischen Identitäten in der deutschsprachigen Nachkriegsmedizin eine bedeutende Rolle spielten, die aber heute weithin und zu Unrecht in Vergessenheit geraten sind. Das breite Spektrum von Begründungsmöglichkeiten klinisch-wissenschaftlicher Evidenz, die Komplexität, Tiefe und Persistenz der Debatten zur Forschungsmethodologie der klinischen Medizin, die Vielfalt der eingenommenen Positionen und weiterführenden Forschungsinitiativen bieten Bezugspunkte und Reflexionspotenziale auch für aktuelle Diskussionen über Formen der Wissenschaftlichkeit in der Medizin.

## Footnotes

1 Auf den Wiesbadener Internistenkongress und seine Bedeutung für die Geschichte der Psychosomatischen Medizin ist bereits von verschiedener Seite hingewiesen worden (siehe u. a. Bormuth 2002; Geisthövel & Hitzer 2019), ohne jedoch auf den Kontext und seine epistemologischen Herausforderungen näher einzugehen.

2 Die Artikel basieren auf der gleichnamigen Sektion auf der 3. Jahrestagung der Gesellschaft für Geschichte der Wissenschaften, der Medizin und der Technik (GWMT) in Bonn (Rahmenthema: *Evidenz in den Wissenschaften*), ergänzt um einen Text von Steffen Dörre über Alexander Mitscherlich.
